# COVID-19-related impact on mental health and career uncertainty in student-athletes—Data from a cohort of 7,025 athletes in an elite sport high school system in Sweden

**DOI:** 10.3389/fspor.2022.943402

**Published:** 2022-09-20

**Authors:** Anders Håkansson, Karin Moesch, Göran Kenttä

**Affiliations:** ^1^Department of Clinical Sciences Lund, Psychiatry, Faculty of Medicine, Lund University, Lund, Sweden; ^2^Region Skåne, Clinical Sports and Mental Health Unit, Malmö, Sweden; ^3^Swedish Sports Confederation, Stockholm, Sweden; ^4^Department of Sports Sciences, Malmö University, Malmö, Sweden; ^5^The Swedish School of Sport and Health Sciences, Stockholm, Sweden; ^6^School of Human Kinetics, University of Ottawa, Ottawa, ON, Canada

**Keywords:** COVID-19, pandemic, athletes, student-athletes, adolescents, depression, anxiety, gaming disorder

## Abstract

**Objectives:**

Mental health consequences and behavior change has been described in elite athletes following the vast impact of the COVID-19 pandemic on the world of sports. However, most study samples have been of limited size, and few studies have assessed student-athletes. This study aimed to analyze perceived mental health impact, measured as clinical degree of depression and anxiety, worry about one's sport and about one's career, and behavioral change with respect to video gaming behavior, in high-school athletes in Sweden.

**Methods:**

Data on anxiety and depression as well as on perceived behavioral changes during COVID-19 were collected from students at sports high schools in Sweden (*N* = 7,025) in February 2021, during the ongoing COVID-19 pandemic.

**Results:**

Sixteen and 14% met criteria of moderate/severe depression and anxiety, respectively. Many respondents reported feeling mentally worse during the pandemic (66%), and were worried about the future of their sport (45%) or about their own future in sports (45%). Increased gaming behavior during COVID-19 was reported by 29%. All mental health variables were significantly more common in women, except increased gaming (more common in men). Being worried about one's career was less common in winter sports, more common in team sports and more common in older student-athletes, and associated with both depression and anxiety in regression analyses.

**Discussion:**

Self-reported mental health impact of COVID-19 is substantial in student-athletes, and even more so in women and in team sports. The lower impact in winter athletes suggests a moderating effect of the seasons in which the COVID-19 outbreak occurred.

## Introduction

During the past few years, increased attention has targeted the need for intervention programs, prevention and treatment for poor mental health in high-performance athletes (Moesch et al., 2018; Reardon et al., 2020; Vella et al., 2021). Moreover, specific concerns have been raised about athletes' mental health during the COVID-19 pandemic, such as in their preparation for the Olympic and Paralympic Games, or otherwise their situation during sports lock-down and uncertainty about future sports events and their career (Håkansson et al., 2020; Samuel et al., 2020; Cardinale, 2021; Stambulova et al., 2022). Mental health in elite athletes has been shown to worsen during the early phases of confinement due to COVID-19 (Mehrsafar et al., 2020). Recently, a systematic review demonstrated that professional athletes aged over 18 years had been negatively affected in their mental health status, due to the changes occurring during the pandemic, typically with a higher impact on mental health in women than in men (Jurecka et al., 2021). Noteworthy, while lockdown and associated lifestyle changes have affected the whole population, it has been suggested that athletes may be at least at the same risk of developing mental distress as the general population, and that a strong athlete identification might even be a risk factor of particularly high anxiety as a reaction to COVID-19 changes (Knowles et al., 2021). Altogether, there is a need to further examine, in large study samples, the subjective effects and mental health in athletes following the immense changes in everyday lives during the pandemic.

So far, less research has focused on mental health in young athletes, such as high school or college athletes, during COVID-19 (Batalla-Gavalda et al., 2021; Fitzgerald et al., 2021; McGuine et al., 2021; Watson and Koontz, 2021). Of particular importance, it has been highlighted that student-athletes already before the pandemic were at high risk of poor mental health, calling for particular attention to this group in light of the pandemic situation (Grubic et al., 2021). Further, it has been postulated that a large proportion of mental health disorders derives from early manifestations of mental ill-health already during adolescence (Jones, 2013), which underlines the importance of studying the impact of the COVID-19 pandemic specifically on adolescent elite athletes.

Competitive events and training routines, both on a professional level and at amateur levels, involved extensive adaptations and thorough testing procedures at all age levels (Krug et al., 2021). Moreover, among young athletes in school settings, transmission of the SARS-CoV-2 virus has been highlighted, possibly with specific risk factors related to the everyday lives of student-athletes (Teran et al., 2020), although other data has indicated that student-athlete transmission of COVID-19 may reflect the epidemiological situation in each region, rather than transmission related to sports events specifically (Sasser et al., 2021). Regardless of this, everyday lives of student-athletes have been clearly adapted and undergone significant change due to COVID-related restriction, and in addition, it can be assumed that the changes to the world of sports overall, including young athletes' career opportunities, may have affected student-athletes to a major extent. In addition, given the seasonality in SARS-CoV-2 transmission, with an increased incidence in the winter season (Johns Hopkins University, 2021), it can also be suspected that some sports strongly related to specific seasons, such as typical winter sports, may be at risk to be more severely affected by societal changes during COVID-19. For example, in the setting studied here, virus transmission and restrictions in society increased markedly in the winter season of 2020/2021, after a marked decrease in virus impact on society during the summer months of 2020 (Public Health Agency).

A number of behavioral changes have been suspected to occur during the pandemic. One of the changes potentially occurring in young people during the pandemic is related to increased online behavior including increased, and more problematic, video gaming behavior (Xu et al., 2021). Gaming disorder, recently recognized as a diagnostic entity (World Health Organization, 2018), is one of the most common addictive conditions in the population, in particular among the young (Stevens et al., 2021), and it is also one of the lifestyle behaviors addressed in the COVID-19 literature as a potentially negative consequence of spending more time at home and due to other changes in working life or school conditions (Amin et al., 2020; King et al., 2020; Claesdotter et al., 2021). Recent population data from the setting assessed in the present paper, Sweden, demonstrated a link between increased self-reported gaming behavior during COVID-19 and poor mental health (Claesdotter et al., 2021).

Problematic gaming behaviors or a fully developed gaming disorder are health hazards which are less addressed in the world of sports. However, negative health effects from gaming behavior in athletes have been suggested (Wattanapisit et al., 2021), and media reports have highlighted an increasing trend of video gaming behavior in young athletes (Washington Post, 2018), including gaming as a specific consequence of lifestyle changes during the COVID-19 pandemic (Washington Post, 2020). Thus, there is reason to examine how gaming behaviors may have changed in high school athletes during the pandemic. In addition, the literature on problem gaming in athletes in general is hitherto sparse (Håkansson et al., 2021).

Altogether, limited research has sampled young student-athletes with attention to mental health during the COVID-19 pandemic (Batalla-Gavalda et al., 2021; Conde et al., 2021; Garver et al., 2021; McGuine et al., 2021). Therefore, the present study aimed to examine mental health parameters in a cohort of student-athletes, within a nation-wide elite sport high school system in Sweden, during the COVID-19 pandemic, including the subjective impact on mental health and worry about the future of their sports and their own future in their sport, clinical ratings of depression and anxiety, and behavioral changes related to video gaming behavior. More specifically, based on previous research from our setting and the nature of the pandemic situation (Håkansson et al., 2020), we hypothesized that mental health impact may be different in winter vs. non-winter sports, in team vs. individual sports, between women and men, and may be larger in older adolescents closer to an adult career planning, and finally we also hypothesized that measures of a clinical-level mental health impact would be associated with the perceived impact on one's sports career.

## Methods

### Study procedures

The present study is an online, anonymous survey, addressing student-athletes within an elite sport high school system in Sweden. The system of sports high schools in Sweden includes about 1,200 student elite athletes selected by a national application system, and approximately another 10,000 student elite athletes selected by a regional and local application system. Once annually, the Swedish Sports Confederation carries out a survey with these athletes, in order to examine factors such as lifestyle and wellbeing, school and sports satisfaction and career planning. The present study added a number of questions related to psychological distress and changes related to COVID-19. This survey was introduced at the end of the annual service, addressing the totality of students at the sports high schools in the country, and these study items were introduced by a written information to the participants, and questions were opened only after the provision of an electronic informed consent. In addition to the study items, separate demographic items were derived from the original annual survey, in line with the information given to participants.

This study was carried out in February, 2021. At that time, SARS-CoV-2 virus transmission was substantial in virtually all parts of Sweden; after a first severe phase of disease during the spring months of 2020, affecting some regions more than others, from October/November, 2020, a significant second wave hit Sweden overall and was still significant at the time of the present study (Johns Hopkins University, 2021). During that period, restrictions in practice were applied during some weeks for athletes above 15 years of age who still are not professional athletes, and thus involved participants in this research.

The study was reviewed by the Swedish Ethics Review Authority (file number 2020-07246), which decided that the present study did not involve personal data which can be linked to an identified individual, and therefore, the study did not formally require approval under the research in ethics act.

### Participants

A total of 8,441 individuals responded to the main survey, and among them, 7,030 provided informed consent and continued to the present study. Five of these individuals did not answer the first questions of the study survey after the consent, and with few exceptions none of the following items, and were therefore excluded. Thereby, a final sample of 7,025 were included in the study.

Participants represented a broad range of 48 different sports, the most common being football (25%, *n* = 1,750), handball (14%, *n* = 1,018), floor ball (13%, *n* = 901), basketball (7%, *n* = 487), golf (4%, *n* = 267), cross-country skiing (4%, *n* = 248), athletics (3%, *n* = 242), orienteering (3%, *n* = 203), ice hockey (3%, *n* = 191), alpine skiing (3%, *n* = 182), bandy (2%, *n* = 171), swimming (2%, *n* = 168), volleyball (2%, *n* = 121), equestrian sports (2%, *n* = 119), tennis (1%, *n* = 100), bicycle (1%, *n* = 86), badminton (1%, *n* = 76), and gymnastics (1%, *n* = 68).

### Variables

From the annual survey, data were derived regarding gender (woman, man), and type of sport. Less frequent sports are not reported separately in the present study, in order to ensure confidentiality. Year in school (1–4) was reported as a proxy for age. In the sports high school system, the fourth year is optional for students choosing a lower study pace, and therefore includes a lower number of respondents.

The following variables were collected in the study:

- Depressive symptoms [Patient Health Questionnaire, PHQ-9 (Kroenke et al., 2001) and symptoms of anxiety [GAD-7 (Spitzer et al., 2006)]. PHQ-9 contains nine and GAD-7 seven items, all coded from 0 to 3, ranging from having experienced the respective symptom during the last 14 days between “not at all” to “almost every day” (with a total score of 0–27 and 0–21, respectively). As recommended in Kroenke et al. (2001) for the PHQ-9 and Löwe et al. (2008) for GAD-7 and as used in previous studies in the elite-sport context with the same questionnaires (Åkesdotter et al., 2020; Håkansson et al., 2020), for both scales, a score of 5 or more was considered to represent mild depressive/anxiety symptoms, 10 or more was considered to represent moderate depressive/anxiety symptoms, and 15 or more was considered severe in the present study.
- Behavioral changes during COVID-19. Here, a brief introduction summarized the COVID-19 situation and the fact that is has had an impact on work, school, leisure activities, and sports in Sweden, and respondents were asked about potential changes “since these changes in Sweden started”:
- Self-reported change in psychological health due to COVID-19 (whether the respondent is feeling psychologically much worse, slightly worse, better, or unchanged). In order to facilitate comparisons, this question was derived from the previous study related to the COVID-19-specific situation in elite athletes in Sweden (Håkansson et al., 2020).
- Self-reported worry about the future of one's sport in Sweden (very worried, slightly worried, not worried, or neither), derived from Håkansson et al. (2020).
- Self-reported worry about one's own career in sports (very worried, slightly worried, not worried, or “I have lost motivation and thought about ending my career due to the current crisis”), derived from Håkansson et al. (2020).
- Changes in gaming behavior during COVID-19, defining this as games to be played on different platforms, including gaming consols, mobile telephones or tablets, and which are not aimed for winning real money (play more games during COVID-19 than before, less than before, unchanged, or “I don't play games, neither now nor before”). The question is an adaptation from a question about gambling for money in previous published studies, such as in the study in elite athletes (Håkansson et al., 2020) and in a study in the general population (Claesdotter et al., 2021), and identical to questions about gaming behavior during COVID-19 currently analyzed in hitherto published research.
- Type of sport. Here, sports were categorized into team sports vs. individual sports (three individuals reporting para sport of the deaf were categorized as non-team sports, i.e., individual sports). In a secondary analysis, sports were categorized into winter sports (ice hockey, bandy, curling, figure skating, and all sports related to skiing) vs. all others.

### Statistical methods

Self-reported mental distress due to COVID-19, worry about the future of the sport in Sweden and about one's own future in sports, and increased gaming behaviors, as well as the endorsing of moderate depression or anxiety, were compared for females vs. males, for participants in team sports vs. individual sports, and for each of the 4 years in school (corresponding approximately to year of age). In addition, self-reported mental distress and worry about one's sport and about one's own career were compared for participants in winter sports vs. other sports. All these calculations were made using chi-square tests (linear-by-linear for the four categories of years in school/age).

Logistic regressions were performed with feeling worse during COVID-19, worry about one's sport, worry about one's own future career in sports, increased gaming behaviors, moderate depressive symptoms and moderate anxiety, respectively, as outcome variables. Gender, years in school, and type of sport (individual vs. team) were included as independent variables. In sub-analyses regarding the potentially larger impact of COVID-19 on winter sports, due to the widespread virus transmission during that period, it was hypothesized that the measures of distress related to the COVID-19 situation in sports would be worse in winter sports athletes. Therefore, the regression analyses measuring the aspect of feeling worse during COVID-19, worry about one's sport and worry about one's career, were carried out with adjustment for winter sports (vs. others) instead of adjusting for team vs. individual sports.

In addition, as one's own situation in sports may potentially affect one's own mental health, in the logistic regression analyses for depression and anxiety, secondary analyses added the item of being worried about one's own career. Overall, in logistic regression analyses, being slightly or very worried about one's sport and one's career were collapsed into one variable.

All analyses were carried out in SPSS, version 25.0. For all analyses, associations with a *p*-value below 0.05 were considered significant. Logistic regression analyses were reported using odds ratios (OR) with 95% confidence intervals and corresponding *p*-value.

## Results

### Sample characteristics

Fifty-seven% (*n* = 3,989) were men, 43% (*n* = 3,007) were women, and 0% (*n* = 18) reported other or preferred not to answer. Thirty-six percent (*n* = 2,506) were in the first of the four school years, 35% (*n* = 2,435) in the second year, 27% (*n* = 1,875) were in the third year, and 3% (*n* = 204) in the last year (whereas the year in school was missing for five individuals). Sixty-seven percent (*n* = 4,721) belonged to a team sport, and the remaining 33% (*n* = 2,304) belonged to individual sports. A total of 14% (*n* = 1,010) represented a winter sport.

### Symptoms of depression and anxiety and their correlates

Moderate to severe depression, and moderate to severe anxiety, were fulfilled by a total of 16% (*n* = 1,100) and 14% (*n* = 965), respectively. Endorsing a GAD score of 10 or above, in logistic regression (*n* = 6,995), was associated with both female gender [OR 2.72 (2.36–3.13), *p* < 0.001] and increasing year in school [OR 1.19 (1.10–1.29), *p* < 0.001], but not with the type of sport (*p* = 0.16). When adding the variable describing worry about one's own career in sports (*n* = 6,830), GAD score remained significantly associated with female gender and with increasing year in school (but not with the type of sport), as well as with worry about one's own career [OR 2.51 (2.16–2.92), *p* < 0.001].

Endorsing a PHQ score of 10 or above, in logistic regression (*n* = 6,995), was associated with both female gender [OR 2.12 (1.86–2.42), *p* < 0.001], increasing year in school [OR 1.26 (1.17–1.36), *p* < 0.001], and negatively associated with belonging to a team sport [OR 0.83 (0.72–0.95), *p* < 0.01]. When adding the variable describing worry about one's own career in sports (*n* = 6,830), PHQ score remained significantly associated with female gender and with increasing year in school, and negatively associated with team sport, as well as with worry about one's own career [OR 2.22 (1.93–2.55), *p* < 0.001].

### Behavioral changes during COVID-19 and their correlates

Sixty-six percent (*n* = 4,626) reported that they were feeling psychologically worse during the pandemic situation as compared to before. Forty-five percent (*n* = 3,155, missing data in seven individuals) were worried about the future of their sport in Sweden related to the COVID-19 situation, whereas 45% (*n* = 3,075, missing data in 167 individuals) were worried about their own career future in sports due to the current situation. A total of 29% (*n* = 2,021, missing data in four individuals) reported increased gaming during COVID-19.

Associations between each of the outcome variables, in relation to gender, year in school, and type of sport (team vs. individual) are reported in Table 1.

**Table 1:** Variables associated with psychological distress and self-reported change in gaming behavior in high school athletes (*N* = 7,025).

	**Feels worse mentally, % (*n*)**	**Worried about future of the sport, % (*n*)**	**Worried about one's career in sports, % (*n*)**	**Increased gaming, % (*n*)**	**PHQ moderate or higher, % (*n*)**	**GAD-7 moderate or higher, % (*n*)**
**Gender**
Female	74 (2,232)	50 (1,513)	50 (1,472)	11 (341)	21 (643)	20 (613)
Male	60 (2,375)^***^	41 (1,632)^***^	41 (1,592)^***^	42 (1,672)^***^	11 (450)^***^	9 (342)^***^
**Year of school**
1	60 (1,504)	44 (1,091)	42 (1,040)	27 (688)	13 (316)	12 (289)
2	67 (1,636)	47 (1,136)	48 (1,134)	30 (741)	15 (376)	14 (342)
3	71 (1,340)	45 (843)	45 (819)	29 (548)	19 (365)	16 (300)
4	71 (144)^***^	41 (84)^a^	42 (81)^b^	22 (44)^c^	21 (43)^***^	17 (34)^***^
**Sport**
Team	67 (3,161)	47 (2,209)	46 (2,132)	32 (1,493)	14 (677)	13 (610)
Individual	64 (1,465)^**^	41 (946)^***^	42 (943)^***^	23 (528)^***^	18 (423)^***^	15 (355)^**^
**Type of sport**
Winter	63 (636)	43 (429)	41 (208)	25 (257)	15 (148)	14 (139)
Other	66 (3,990)^*^	45 (2,726)	45 (2,667)^*^	29 (1,764)^*^	16 (952)	14 (826)

* *p* < 0.05.

** *p* < 0.01.

*** *p* < 0.001.

a *p* = 0.59, linear-by-linear.

b *p* = 0.08, linear-by-linear.

c *p* = 0.71, linear-by-linear.

Feeling worse mentally during COVID-19, in logistic regression (*n* = 6,995), was associated with both female gender [OR 2.01 (1.81–2.23), *p* < 0.001], increasing year in school [OR 1.30 (1.23–1.38), *p* < 0.001], and belonging to a team sport [OR 1.31 (1.17–1.46), *p* < 0.001]. Being worried about one's sport in Sweden, in logistic regression (*n* = 6,988), was associated with female gender [OR 1.49 (1.35–1.64), *p* < 0.001] and with belonging to a team sport [OR 1.32 (1.19–1.46), *p* < 0.001], but not with increasing year in school (*p* = 0.22). Feeling worried about one's own career in sports, in logistic regression (*n* = 6,830), was associated with both female gender [OR 1.49 (1.35–1.64), *p* < 0.001], increasing year in school [OR 1.07 (1.01–1.13), *p* = 0.02], and belonging to a team sport [OR 1.25 (1.13–1.39), *p* < 0.001]. Reporting increased gaming during COVID-19, in logistic regression (*n* = 6,991), was negatively associated with female gender [OR 0.18 (0.16–0.20), *p* < 0.001], and associated with belonging to a team sport [OR 1.48 (1.31–1.67), *p* < 0.001], but not with increasing year in school (*p* = 0.22).

### Role of winter sports

In the analysis of winter sports vs. others, belonging to a winter sport was negatively associated with feeling worse during COVID-19 (63 vs. 66%, *p* = 0.04), and with being worried about one's own career (41 vs. 45%, *p* = 0.01), and not significantly associated with being worried about the future of the sport overall (43 vs. 45%, *p* = 0.09).

When controlling for winter sport instead of team vs. individual sport, the associations between feeling worse during COVID-19 and female gender and year in school remained, whereas winter sport in itself was marginally and negatively associated with feeling worse (*p* = 0.054). Being worried about one's sport remained associated with female gender and still was not associated with year in school, and also was not associated with winter sport (*p* = 0.29). Being worried about one's own career remained associated with female gender and increase year in school, and was negatively associated with belonging to a winter sport [OR 0.86 (0.75–0.99), *p* = 0.03].

## Discussion

The present study provides a picture of COVID-19-related impact on mental health in a large dataset—larger than in a majority of comparable studies—of high school athletes in Sweden. It confirms, in young athletes, the picture described in often older elite athletes, that a large proportion of athletes feel psychologically affected by COVID-19-related changes, and that symptoms of depression, anxiety and worry about one's own future in sports, describing a subjective impact from COVID-19, are more common in female than in male athletes. Psychological distress such as worry about one's sport and one's own career were more common in team sports as compared to individual sports, whereas winter sports were not associated with a more severe impact. Even when controlling for gender, age, and type of sports, reaching a moderate level of clinical depressive or anxiety symptoms were both independently associated with a worry about one's own career in sports. Increasing video gaming behaviors, a measure of behavioral change that may occur in everyday life of young people during the pandemic, was common in males and in team sports. Altogether, the present study confirms that subjective mood changes and worry about the future of one's career in sport is common in the COVID-19 pandemic, and highlights the need to address such concerns in clinical settings and in the school setting of young athletes.

Overall, high proportions of the student-athletes reported being slightly or very worried about their own career and about their sport related to the COVID-19 situation, and reported feeling psychologically worse during the pandemic. Based on our findings it is assumed that young student-athletes may have a particular risk of having experienced negative emotions in relation to COVID-19, in comparison to their peers (i.e., students not being athletes). For example, in a longitudinal study in Swedish teenagers in general, following both respondents who were exposed, and who were not exposed, to the COVID-19 pandemic, it was demonstrated that stress, relationship concerns and psycho-somatic concerns were not more severe in adolescents who experienced the COVID-19 period (Chen et al., 2021). Thus, mental health consequences in adolescents in the general Swedish population may not be substantial, as reported in other research (Johansson et al., 2021). In light of this, it is of importance to acknowledge that a substantial percentage of young student-athletes endorsed symptoms of increased psychological symptoms during the pandemic. While this is a measure specifically related to an ongoing COVID-19 situation, it remains uncertain how to interpret these numbers and what they would show in any other situation relating to a period of career uncertainty or any crisis occurring to one's sport setting. However, with this in mind, the figures demonstrate the need to maintain vigilance regarding young student-athletes' mental health and uncertain feelings about the future, during the pandemic and beyond. A recent review on mental health in student athletes stressed the importance to use validated measures when examining mental ill-health, such as depression and anxiety, and also to continue to examine the impact of COVID on mental health (Kegelaers et al., 2022). Stakeholders of the Swedish elite sport high school system have raised concerns for an increase in mental health problems among the student-athletes investigated. Scholars have argued that this increased attention to mental health in elite sports also requires guidelines and framework to respond to these needs (Purcell et al., 2019). Vella and colleagues (Vella et al., 2021) critically discussed the lack of involvement and endorsement from national sports organizations in their review of thirteen published position statements for mental health policy in sport. In contrast, the Swedish Sport Confederation has taken an active role to lead and develop the high school system ever since the start in late 1960's with attention to study and sports results and more recently also with attention to manage mental health by prompting research, developing specific resources and support (e.g., a webpage and video files with psychoeducation and exercises).

Results from this study can be compared to the data from a previous study in the same setting (i.e., in Sweden), but collected from adult elite athletes in the top leagues of three major team sports in Sweden, soccer, ice hockey, and handball (Håkansson et al., 2020). In the present study, among the team sports participants, 67% reported feeling psychologically worse during the pandemic. In contrast, among adult elite athletes, 52% reported feeling worse during the pandemic. Although these figures are difficult to compare, among other because of possible gender differences, at least it appears that self-reported psychological impact from the pandemic situation may at least not be smaller in high school athletes, than in their adult elite level counterparts who are somewhat older. In the present high school study, a total of 47 and 46% reported being worried about their sport and about their own career. This corresponds to a total of 66 and 51%, respectively, who reported being worried about their sport and about their own career, among adult elite team athletes. Thus, high school athletes may be somewhat less worried about the sports future than elite athletes, and this may potentially be due to the fact that elite athletes, often professional or semi-professional, may in short term be more affected by the situation of their sport, compared to high school students who may perceive some distance to an adult elite career phase where they potentially depend more financially on their sport. Likewise, student athletes may not be as close to the peak of their sports career, compared to elite athletes, and may feel less distress due to that. In contrast, however, figures describing worry about one's own career differed little between team athletes in the two studies.

Being a team sports athlete was associated with feeling worse during the pandemic situation, with worry about one's sport overall, and worry about one's own career. While virtually all sport categories have been affected by the COVID-19 pandemic, team sports are among those more severely affected during the early phases of the pandemic (Håkansson et al., 2020). Also, even after the return to play of team sports in many countries, although with reduced or no audience, team athletes may have had further reason to worry about their ongoing career and about their sports and future financing. For example, in early phase of confinement during COVID-19, it has been reported that team sports athletes have reported more negative impact than individual athletes, and that athletes in individual sports felt more able to cope with challenges related to the pandemic situation (Martínez-Patiño et al., 2021). In a range of sports audience was absent due to lock-down procedures or other restrictions, major international tournaments were canceled or postponed, and also, but more frequently in team-sports, virus outbreaks in athletes have repeatedly forced team sports into cancellations or substantial adaptations. For example, in the setting studied here, even after the return to the 2020/2021 season in elite Swedish ice hockey, COVID-19 outbreaks within teams caused major disruptions to the season (Reuters, 2020), and as an example, the Junior World Cup ice hockey tournament was even canceled during the event, due to COVID-19 outbreaks (New York Times, 2021). However, the research in this area is still to sparse too fully explain these findings, but these major challenges seem to impact team sport athletes and their mental health to a larger extent.

Nevertheless, it cannot readily be concluded that differences between team sports and individual sports would be the same in a country applying less mandatory COVID-related restrictions, such as Sweden, as in countries with clear lockdown procedures. Indeed, the reporting of lifestyle changes and mental health impact has in some literature focused on proper lockdown procedures (Martínez-Patiño et al., 2021; Moscoso-Sánchez et al., 2021), which were not implemented in Sweden during any phase of COVID-19. However, in the Swedish setting, one would intuitively assume that individual sports would go less affected by anti-virus recommendations against public gathering which would instead have a substantial impact on team sports and their match events or major tournaments. In contrast, however, other data has indicated that belonging to a team in a team sport was associated with lower levels of pandemic-related distress than in individuals athletes, with the hypothesis that coping would be easier in a social team environment (Fiorilli et al., 2021). Altogether, data in the area is inconclusive, and the impact on athletes in different types of sports, in different settings, and at different stages in their career should be a focus in further research studies in the area.

In the present study, depression and anxiety in women were highly comparable to those of the previous Swedish study in elite soccer, ice hockey and handball players (Håkansson et al., 2020). However, the figures for depression and anxiety in men were higher in the present study compared to the previous elite team sports study (Håkansson et al., 2020). Likewise, in a Swedish study in elite athletes carried out prior to the COVID-19 pandemic (Johansson et al., 2021), figures for depression and anxiety were also comparable to those of the COVID-19-related team sports study; thus, with prevalence rates for men somewhat lower than in the male high school students of the present study. Altogether, the rates of depression and anxiety presented in this study are also within the range comparable to other international studies (Nixdorf et al., 2013; Gouttebarge et al., 2015), although exceptions with markedly lower prevalence figures also exist (Junge and Feddermann-Demont, 2016). However, within the geographical context studied here, it appears that among males, high school athletes may be at a comparably higher risk of reaching a clinical level of mental distress, during COVID-19, than do their (mostly) older counterparts in elite teams. This is consistent with previous research data showing that anxiety levels may be higher in younger athletes than among older athletes (Rice et al., 2019).

Increased depressive states in female athletes, compared to male athletes, have been shown previously, such as in a smaller study in Spanish athletes, among whom a majority were student-athletes (Conde et al., 2021), in a relatively large study of elite athletes in Spain, where depression rates were also significantly higher in women (Jaenes Sánchez et al., 2021), and in a large study of US adolescents (McGuine et al., 2021). Also, in comparison to a recent Norwegian study in elite athletes (Pensgaard et al., 2021), rates of depression in the present study were comparably high in females but markedly lower in males; in the present high school study, gender differences were large, with the prevalence of depression and anxiety being roughly twice as high in women as in men. Overall, anxiety rates were lower in the Norwegian elite athlete study than in the present one. Thus, gender differences were pronounced in the high school setting, but also comparable to the previous Swedish elite team study (Håkansson et al., 2020) and other studies, where anxiety is markedly higher among female than among male elite athletes (Rice et al., 2019).

Likewise, women were more likely to feel worse during the pandemic, and more likely to worry about one's sport and about one's own career. Women may, in many sports, face more difficult financial conditions, including more unstable semi-professional situations where the income relies, to a very large extent, on a different professional career (i.e., outside of sport), and these factors may amplify the challenges followed by the pandemic situation (Bowes et al., 2020). Here, only high school students were assessed, such that the current occupational and personal financial situation formally should not differ between women and men, but young athletes' anticipation of future financial conditions is likely to differ between women and men. Altogether, this may put women into more of a financial uncertainty situation as compared to men. Gender differences are in line with the previous study in elite athletes in the same setting (Håkansson et al., 2020) and in others, where female athletes have reported a higher mental health impact from the pandemic than male athletes do (Pillay et al., 2020; Pons et al., 2020; di Fronso et al., 2022). Also, in line with this, other data have indicated that during COVID-19 confinement, there was a closer association between mental health impact and sports and performance-related variables in female athletes, compared to male athletes (Mon-López et al., 2020).

Older age, i.e., being closer to one's professional post-high-school career, was associated with worry about one's own career, but not with worry about the sport overall. Again, being closer to a future professional or semi-professional career is likely to present a higher degree of uncertainty in a situation where practice opportunities, championships or major qualifying competitions are lost due to the pandemic. For this reason, it appears logical that the greater the time distance to a future post-high school career, the lower the level of insecurity perceived. In addition, athletes' mood in the preparations for uncertain events during the lock-down is likely related to personal factors, where individuals with higher coping capability and better sense of coherence are likely to withstand the pandemic crisis better (Szczypinska et al., 2021).

Winter sports participants overall displayed lower degree of worry about one's own career. Overall, winter sports were not affected during the first phase of the pandemic, which occurred mainly during the spring months of 2020, and the complete sports lock-down periods (Håkansson et al., 2020) had been terminated when winter sports prepared and started their seasons during the autumn of 2020. However, the winter season of 2020/2021 in the present geographical setting was strongly associated with a surge in COVID-19 transmission (Johns Hopkins University, 2021), because of key features demonstrated in the virus itself, making the transmission higher in the colder season (Kumar et al., 2021), and thereby increasing the need for societal restrictions in the winter season. In line with this, virus-preventing strategies in the present setting were re-introduced during the winter season of 2020/2021, after some months of strongly reduced societal measures during the summer and early autumn of 2020 (Public Health Agency). Hitherto, to the best of the authors' knowledge, no studies have assessed the mental health impact in athletes with respect to different seasonal aspects throughout the year. Thus, further studies assessing this particular issue may be needed, but a plausible explanation is probably the fact that the most extensive outbreak of COVID-19, and the introduction of extensive restrictions to society in 2020, occurred at the end or after the end of the 2019/2020 winter season for many sports, such that despite the longstanding COVID-19 impact a year later, winter athletes may have had more time and better possibilities to cope with and adapt to the pandemic.

The hypothesized behavioral change during the pandemic, an increase in video gaming behavior, was markedly more common in men than in women. In addition, team sports athletes were more likely to report increased gaming, even when controlling for gender and age. Gaming in athletes only has been sparsely addressed in prior research. A survey study in Swedish athletes demonstrated low rates of problem gaming in young athletes, and despite low numbers in a relatively limited study sample, problem gaming tended to be more common in male athletes than in their female counterparts (Håkansson et al., 2018). According to general population survey data from the same geographical setting and others, a markedly more extensive gaming behavior in men can be suspected, in comparison to women (André et al., 2020; Stevens et al., 2021). Thus, while the gender difference seen in the present study is far from surprising, it remains to be understood why this behavioral change during COVID-19 (even when controlling for age and gender) was more common in team sports.

Overall, there is a lack of study data on changes in gaming behavior in the general population during the COVID-19 pandemic. Recent data from the same setting, Sweden, in a web-recruited general population sample (Claesdotter et al., 2021), demonstrated that in the youngest age group (16–24 years), as many as 57% of respondents reported an increased gaming behavior during the pandemic, i.e., with the same type of survey variable as in the present study, but within a wider age range. In that study, however, high rates of self-reported increase in gaming behavior (51%) was also seen in the next age category (25–29 years), with markedly lower numbers from age 40 and above. Thus, it can be suspected that rates of increasing gaming behaviors during the pandemic are higher in the youngest age groups, such as those included in the present high school study. Thus, based on this comparison, it seems plausible to conclude that increases in gaming behavior are smaller in this sample of high school athletes, than in a young general population sample. It remains to be studied whether this may be related to adherence of these respondents to high-level sports, or to other features of the population.

Here, it can be hypothesized that personality factors, or possibly group dynamics within the team context in young individuals, may increase gaming behavior. For example, it has been demonstrated in student-athletes that mental health was better in subgroups who perceived a better connectedness to peer athletes, and who maintained their student-athlete identity (Graupensperger et al., 2020). It also appears that the degree of negative impact from COVID-19 varies depending on the context of individuals and how much exercise possibilities were affected (Pons et al., 2020). Otherwise, also, one might consider that this type of behavioral change, occurring in a context of restrictions and lifestyle changes enforced by the pandemic, is yet another example of how team athletes were more emotionally affected by the pandemic changes than individual athletes. Here, more research may be needed in order to confirm possible hypotheses, and mental health in team sports athletes may be particularly important to address in pandemic or similar crises causing a major change in planning and everyday habits.

The present study has a number of implications. In young student-athletes, intervention programs for the remaining phases of the COVID-19 pandemic worldwide, and for the return to normalized sports events after a long time of pandemic-related adaptations, have to address mental health issues, over and above aspects related to physical health and injury prevention. Hitherto, such mental health issues have received less attention and resources in contrast to the traditional health support provided by sports medicine. Nevertheless, stakeholders and sport organizations have developed guidelines for mental health with special attention to the pandemic situation (Stambulova et al., 2022). Also, previous knowledge and initiatives on the promotion of mental health in sports may be particularly important to apply in the present situation. Such previous consensus statements have, for example, included the need to set minimum levels of training for staff involved in mental health management in sports, and to outline guidelines for when to refer clients to specialized care instead of within-sports resources (Breslin et al., 2019). Such initiatives, and such guidelines, may need to include awareness of COVID-19-related issues when seeking to detect and prevent mental health problems. Other implications include the need for allocation of resources to mental health promotion in sports in general; given the gender differences seen here, the challenges of young female athletes may have to be particularly addressed in mental health work within the world of sports. In addition, problematic gaming behavior, only recently addressed in research in elite or student athletes, may need to be highlighted further as a possible co-factor in mental health and behavior in young athletes.

The present study is surrounded by a number of limitations. Some of these are related to its methodological design as a self-report survey, and in an online format, where the number of questions, and extent of longer formalized diagnostic tools, have to be limited. For example, items describing the negative impact from COVID-19 were brief with categorical response options, which limits the nuances of data in comparison to a more in-depth quantitative or qualitative examination. Also, more detailed socio-economic or socio-demographic information, including ethnicity, were not available in the present study. Likewise, the study is cross-sectional, and comparisons over time, such as a follow-up in the same individuals, are therefore not possible. In addition, the generalizability of study findings related to COVID-19 is affected by the fact that different regulations in society have been seen in different countries, and during different phases of the pandemic. Sweden is one of the few comparable countries which never applied an actual lock-down strategy during the pandemic (Baral et al., 2020; Yarmol-Matusiak et al., 2021). Thus, although domestic competitive events in sport in Sweden were entirely canceled for a period in the spring months of 2020, and substantially affected during more prolonged phases in 2020 and 2021, participants in the present study have not faced a home confinement regulation such as that applied in a number of countries during lock-down periods. This includes the fact that despite recommendations and some more mandatory regulations, restaurants, grocery stores, a number of public facilities and training facilities were never closed in Sweden. However, although large parts of the society remained open, the respondents in the present study had faced distance school education from home, and likely a range of cancellations or considerable adaptations to practice in school. For these reasons, with respect to the actual sports situation addressed in the study, it is likely that the impact on these participants may have been substantial and comparable to that of many other countries.

## Conclusions

Psychological distress and self-reported worry about sports is common in high school athletes during the COVID-19 pandemic. This mental health impact was smaller in winter sports, but higher in team sports athletes, among whom subjective mental health consequences appeared to be more common than in previous study data from mostly older elite athletes. Mental health impact, and clinical levels of depression and anxiety, were more common in female than in male high school athletes, and also higher in older high school athletes. Increasing gaming behavior was a relatively common behavioral change during COVID-19, and more so in team sports athletes and in males.

## Data availability statement

The raw data supporting the conclusions of this article will be made available by the authors, without undue reservation.

## Ethics statement

The studies involving human participants were reviewed and approved by Swedish Ethics Authority. Written informed consent from the participants' legal guardian/next of kin was not required to participate in this study in accordance with the national legislation and the institutional requirements.

## Author contributions

AH and GK planned the data collection. AH carried out statistical analyses and wrote the manuscript draft. KM and GK provided comments and editing. All authors contributed to the overall research idea. All authors approved the final version of the paper.

## Conflict of interest

Author AH has funding from the state-owned gambling operator of Sweden, AB Svenska Spel, which is not in any way involved in the present project, and from the research council of the Swedish alcohol monopoly, which also had no role in the present research. The remaining authors declare that the research was conducted in the absence of any commercial or financial relationships that could be construed as a potential conflict of interest.

## Publisher's note

All claims expressed in this article are solely those of the authors and do not necessarily represent those of their affiliated organizations, or those of the publisher, the editors and the reviewers. Any product that may be evaluated in this article, or claim that may be made by its manufacturer, is not guaranteed or endorsed by the publisher.
